# Identification of a binding protein for sesamin and characterization of its roles in plant growth

**DOI:** 10.1038/s41598-019-45003-7

**Published:** 2019-06-14

**Authors:** Masayuki Tera, Tomotsugu Koyama, Jun Murata, Ayako Furukawa, Shoko Mori, Toshiaki Azuma, Takehiro Watanabe, Katsuhito Hori, Atsushi Okazawa, Yasuaki Kabe, Makoto Suematsu, Honoo Satake, Eiichiro Ono, Manabu Horikawa

**Affiliations:** 1 Bioorganic Research Institute, Suntory Foundation for Life Sciences (SUNBOR), 8-1-1 Seikadai, Seika, Soraku, Kyoto, 619-0284 Japan; 2 Graduate School of Engineering, Osaka University, 2-1 Yamadaoka, Suita Osaka, 565-0871 Japan; 3 Graduate School of Life and Environmental Sciences, Osaka Prefecture University, 1-1 Gakuen-cho, Naka-ku, Sakai Osaka, 599-8531 Japan; 4 Department of Biochemistry, Keio University School of Medicine, 35 Shinanomachi, Shinjyuku-ku, Tokyo, 160-8582 Japan; 5 Japan Agency for Medical Research and Development (AMED), Core Research for Evolutional Science and Technology (CREST), 1-7-1 Otemachi, Chiyoda-ku, 100-0004 Japan; 6 Research Institute, Suntory Global Innovation Center Ltd (SIC), 8-1-1 Seikadai, Seika, Soraku, Kyoto, 619-0284 Japan

**Keywords:** Target identification, Plant molecular biology

## Abstract

Sesamin is a furofuran-type lignan that is found abundantly in seeds of *Sesamum indicum* (sesame) and has been widely accepted as a dietary supplement with positive effects on human health. The biological activity of sesamin in human cells and organs has been analysed extensively, although comparatively few studies show biological functions for sesamin *in planta*. Herein we screened sesamin-binding proteins (SBP) from sesame seedling extracts using sesamin-immobilized nano-beads. In subsequent peptide mass fingerprinting analyses, we identified a SBP, Steroleosin B, which is one of the membrane proteins found in oil bodies. In addition, pull-down assays and saturation transfer difference-nuclear magnetic resonance (STD-NMR) experiments demonstrated that sesamin binds directly to recombinant Steroleosin B *in vitro*. Finally, ectopic accumulations of sesamin and Steroleosin B in transgenic *Arabidopsis thaliana* plants induced severe growth defects including suppression of leaf expansion and root elongation. Collectively, these results indicate that sesamin influences tissue development in the presence of Steroleosin B.

## Introduction

Sesame plants have been cultivated for more than 5,000 years to enable their seeds and seed oils to be consumed for foods, fuels and various other applications. Sesame seeds are known to accumulate sesamin and other related furofuran-class lignans, which are a group of polyphenolic plant metabolites derived originally from phenylalanine. The biosynthetic pathway for furofuran class lignans has been well studied and many related enzymes and genes have been identified^1–3^. Two radical molecules of coniferyl alcohol, produced by the phenylpropanoid biosynthetic pathway and then oxidized by unknown mechanisms, are stereo-selectively conjugated by a dirigent protein to produce pinoresinol, which is a central precursor of most lignans^4^.

In sesame seeds, sesamin is synthesized from pinoresinol through the sequential formation of two methylenedioxy bridges in reactions catalysed by the P450 monooxygenase CYP81Q1^5^. Then, a portion of sesamin is oxidatively converted into sesamolin and sesaminol, which generally accumulates as sesaminol triglucosides, by the P450 monooxygenase CYP92B14^6^.

Sesamin has various valuable biological properties in mammals, including anti-oxidative, hypocholesterolemic, anti-inflammatory, neuroprotective and immunomodulatory activities and has been used as an ingredient in health-promoting supplements^1,2^. However, the biological relevance of sesamin to sesame plants themselves remains elusive. For example, sesamin has considerable anti-feedant activities against predators and moderately inhibits the growth of *Spilarctia obliqua*^7^. The putative biological roles of sesamin *in planta* have been associated with seed maturation and/or germination because sesamin content in developing sesame seeds reaches up to 1% (w/v) and decreases drastically upon germination^8^. Collectively, these reports suggest that the endogenous target proteins of sesamin play key roles in seed maturation and/or germination and reflect the biological roles of sesamin *in planta*.

Recently, high performance affinity nano-bead technology has enabled the identification of binding proteins for several small molecules of interest^9^. We therefore used this affinity technique to determine sesamin-binding proteins (SBPs) using the sesamin-immobilized nano-beads (SIB), which led to identification of Steroleosin B^10^ as a candidate protein. Simultaneous ectopic expression of *CYP81Q1* and *Steroleosin B* was shown to retard growth in *Arabidopsis* plants, whereas, no effect was observed when they were expressed individually, implying that sesamin influences plant growth in the presence of Steroleosin B.

## Results

### Identification of Steroleosin B as a sesamin-binding protein

**Figure 1:**
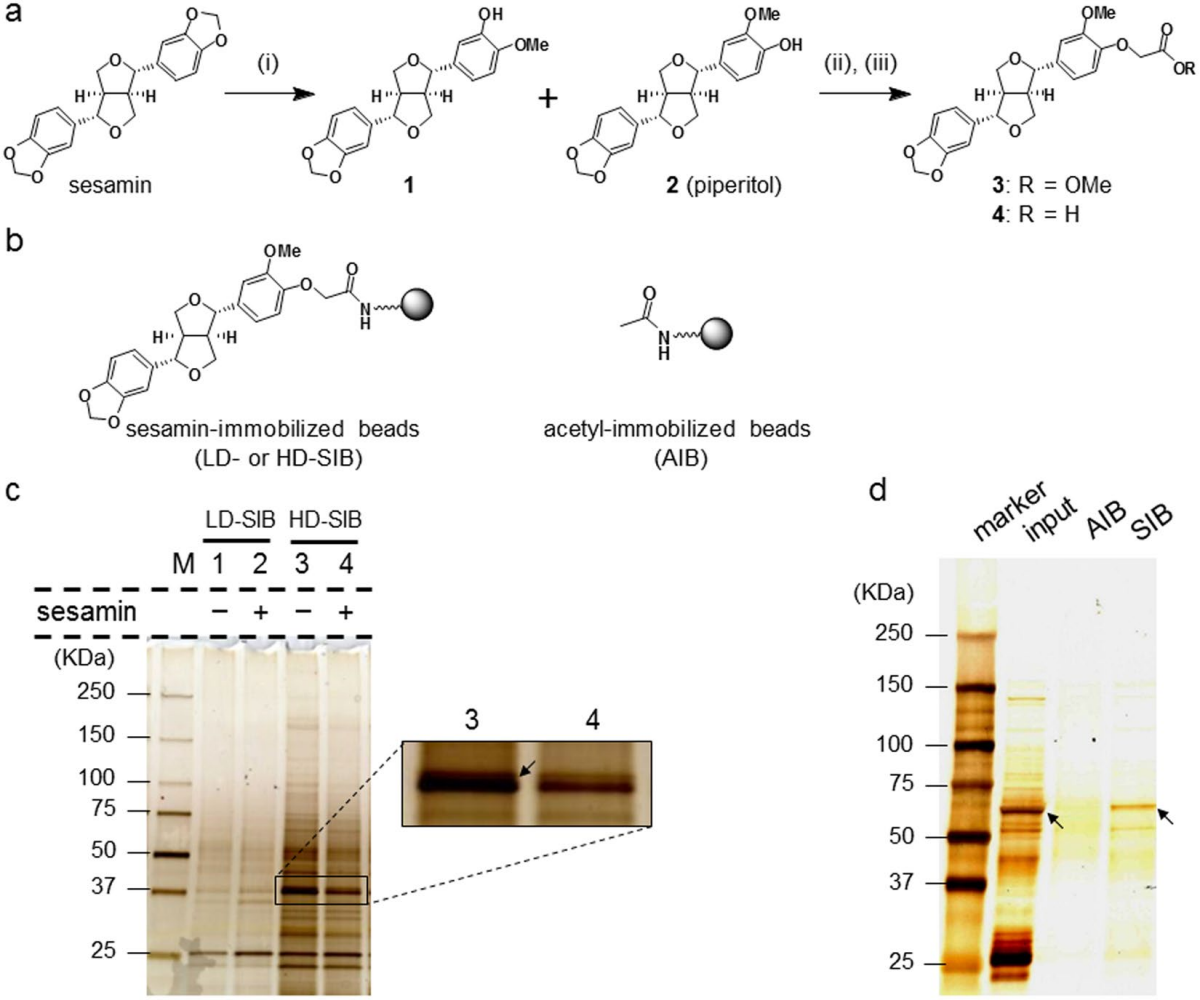
Identification of Steroleosin B as a sesamin-binding protein and purification of recombinant GST-fused Steroleosin B. (**a**) Reaction conditions for the synthesis of sesamin probe **4**; (i) DIBAL, toluene, reflux, 1 h; (ii) BrCH_2_CO_2_Me, K_2_CO_3_, DMF, rt, 1 h; (iii) 1 N NaOH aq., THF, rt, 1 h. (**b**) Structure of LD- or HD-SIB and AIB. (**c**) Lysates from sesame seedlings in the absence (lanes 1 and 3) or presence (lanes 2 and 4) of sesamin were incubated with LD-SIB (lanes 1 and 2) or HD-SIB (lanes 3 and 4). The proteins eluted from SIB were resolved using sodium dodecyl sulfate-poly acrylamide gel electrophoresis (SDS-PAGE) and were then subjected to silver staining. The band marked by the arrow (lane 3 in magnified image) was diminished in the presence of sesamin as a competitor (lane 4). (**d**) Whole cell extracts of *E. coli* producing recombinant GST-fused Steroleosin B were incubated with AIB or SIB. The resulting eluents were resolved using SDS-PAGE followed by silver staining. The arrows in SDS-PAGE show the bands for GST-fused Steroleosin B.

To identify SBPs that are present during sesame seed germination, we performed affinity screening analyses of SBPs using SIB. Initially, we designed and synthesized sesamin derivative **4** (Fig. 1a) as an affinity probe that displays sesamin structures on the surfaces of nano-beads. To this end, sesamin affinity probe **4**, which contains a carboxyl group, was covalently fixed onto amine beads at 0.2 and 2.0 mM to prepare low density- (LD) and high density- (HD) SIB, respectively (Fig. 1b). LD or HD-SIB were then incubated with protein extracts from sesame seedlings in the presence or absence of excess free sesamin. The proteins bound to SIB were then analysed with sodium dodecyl sulfate-polyacrylamide gel electrophoresis (SDS-PAGE) and visualized by silver staining (Fig. 1c). Among the multiple protein bands obtained using HD-SIB, the binding of the band denoted by the arrow was diminished by excess sesamin (Lanes 3 and 4, Fig. 1c), suggesting competitive binding. In-gel digestion and peptide mass fingerprint analysis of corresponding bands identified three proteins from *Sesamum indicum* (Supplementary Tables S1 and S2). Based on Mascot probability scoring^11^, Steroleosin B had the highest score among the candidates. Steroleosin B is one of the membrane proteins known to accumulate primarily in the oil bodies of *S. indicum* and various other plants^10^ that is thought to be involved in the dehydrogenation of hydroxysterol due to the presence of a sterol-binding subdomain. Although Steroleosin B has been shown to metabolize the mammalian steroids estradiol and corticosterone^10^, the endogenous substrates and activities of Steroleosin B *in planta* remain poorly understood. As both sesamin and Steroleosin B highly accumulated in sesame seeds^5,10^, their direct binding analyses were carried out.

### Confirmation of direct interactions between sesamin and Steroleosin B

#### Pull-down assays of recombinant GST-fused Steroleosin B

Since affinity screening only provides candidates that may bind to SIB, verification of their direct interactions is needed. To verify binding between sesamin and Steroleosin B, we performed pull-down experiments using recombinant GST-fused Steroleosin B^10^ and SIB. The resulting SDS-PAGE showed that GST-fused Steroleosin B was captured by SIB similar to the affinity screening. By contrast, Steroleosin B was not captured by control beads (acetyl-immobilized) (AIB, Fig. 1b), indicating that GST-fused Steroleosin B binds to SIB via sesamin-displaying moieties (Fig. 1d).

#### Saturation transfer difference-nuclear magnetic resonance (STD-NMR) analyses of sesamin and recombinant GST-fused Steroleosin B

**Figure 2:**
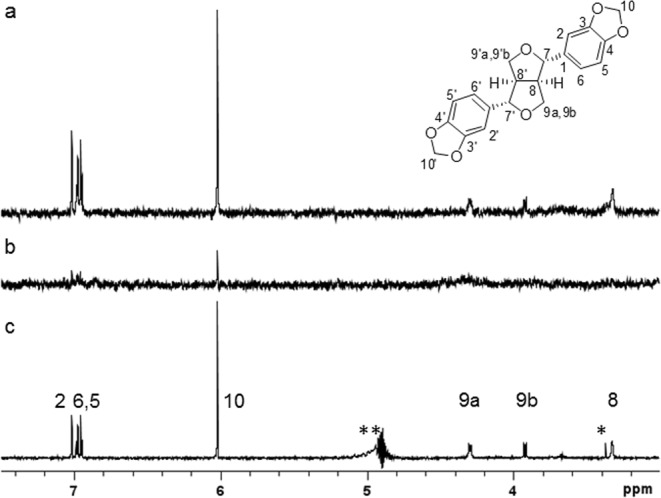
STD-NMR analyses of sesamin and GST-fused Steroleosin B. ¹H STD-NMR spectra of sesamin with GST-fused Steroleosin B (**a**) or GST (**b**) at a ligand to protein molar ratio of 60:1; (**c**) one-dimensional ¹H NMR spectra of sesamin. Signals were assigned to the sesamin structure. Those of C2′, C5′, C6′, C8′, C9′ and C10′ overlapped exactly with those of C2, C5, C6, C8, C9 and C10, respectively, due to the C_2_ symmetric structure of sesamin; *impurity; **C7- and C7′-protons in this area disappeared due to the suppression of non-deuterated water protons with similar chemical shifts.

To confirm the direct interactions between Steroleosin B and sesamin without modification, STD-NMR^12^ was performed to create epitope maps of ligands binding to proteins. STD-NMR spectra for sesamin and GST-fused Steroleosin B (Fig. 2, Supplementary Fig. S1) at a molar ratio of 60:1 were identified in all assigned proton signals of sesamin, except for H-7 and H-7′, which had a signal that overlapped with that of water. Signal intensities of protons (H-2, 2′, H-5, 5′ and H-6, 6′) in aromatic ring moieties, including those (H-10, 10′) in 1,3-methylenedioxy groups, were higher than those (H-8, 8′ and H-9ab, 9′ab) in furofuran ring moieties. This finding showed that the aromatic ring moieties and not the furofuran rings of sesamin were proximately located in Steroleosin B during binding (Fig. 2a). By contrast, STD-NMR spectra from the 60:1 mixture of sesamin and GST showed limited signals from protons of sesamin (Fig. 2b). Taken together with the pull-down analysis, these results demonstrate the direct interaction between sesamin and Steroleosin B.

### *Steroleosin B* is expressed during seed development and post-germination stages

Simultaneous regulation of *Steroleosin B* and *CYP81Q1* in sesame plants is necessary to achieve interactions with Steroleosin B and sesamin. To investigate the regulatory mechanisms for *Steroleosin B* expression in sesame plants, we performed reverse transcriptase-polymerase chain reaction (RT-PCR) analyses using total RNA samples from seeds at various stages of development (Supplementary Fig. S2). The resulting data showed that *Steroleosin B* mRNA is expressed throughout the development of sesame seeds. *CYP81Q1* mRNA, which encodes a sesamin synthase, was detected in all stages of seed development^5^. In addition, *Steroleosin B* and *CYP81Q1* were both expressed during seed germination stages regardless of light conditions, indicating that *Steroleosin B* expression coincides with that of *CYP81Q1* during most of the stages of seed development and germination stages in sesame plants.

### Ectopic accumulation of sesamin and Steroleosin B in transgenic *Arabidopsis* plants results in drastic changes in morphology

We determined whether the functions of sesamin *in planta* are dependent on Steroleosin B because sesamin interacted with Steroleosin B *in vitro*. Specifically, we investigated phenotypic changes in plant physiology and morphology in the presence of both sesamin and Steroleosin B. *Arabidopsis thaliana* plants do not accumulate sesamin but they do contain the precursor pinoresinol and are free from the background effects of endogenous sesamin. In addition, experimental tools for genetic analyses have been well developed in *Arabidopsis* plants, whereas transformation and gene-knockout techniques are not practical in sesame plants. Moreover, we presumed that external administration of sesamin would not be effective in plant bioassays due to sesamin’s poor water solubility.

**Figure 3:**
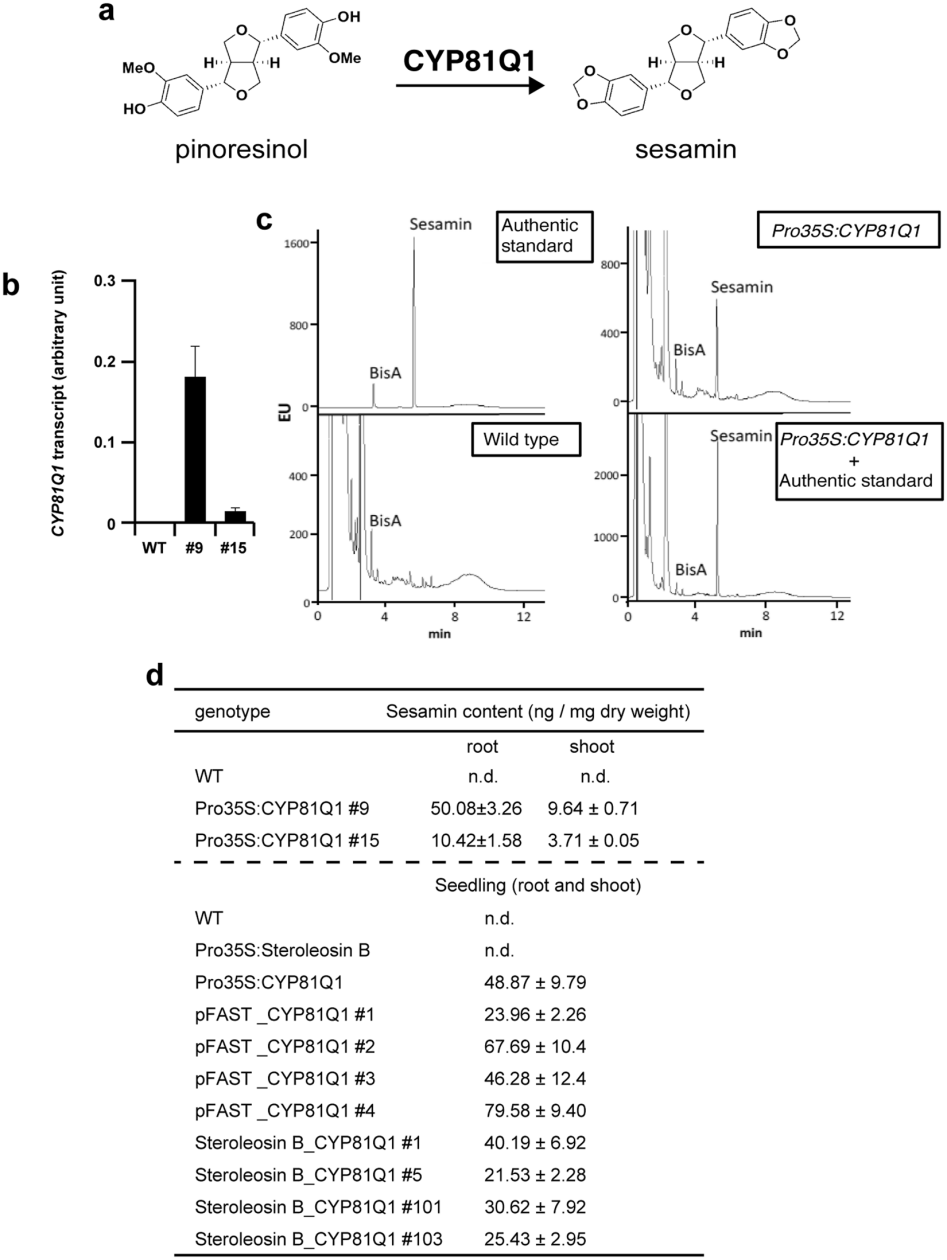
Accumulation of sesamin in *Pro35S:CYP81Q1* and *Pro35S:Steroleosin B/Pro35S:CYP81Q1 Arabidopsis* plants. (**a**) The biosynthetic reaction of CYP81Q1. (**b**) Expression of *CYP81Q1* in *Pro35S:CYP81Q1 Arabidopsis* plants. The *CYP81Q1* level was normalized by the value of *ACTIN2*. Error bars indicate the standard variation (n = 3). (**c**) The florescence due to sesamin from the authentic standard, and the extracts of wild-type and *Pro35S:CYP81Q1 Arabidopsis* plants in the UPLC analysis. The overlapped peak with the same retention time was detected in the sample of *Pro35S:CYP81Q1 Arabidopsis* plants with the addition of the authentic standard (Pro35S:CYP81Q1 + authentic standard). (**d**) Quantification of sesamin in *Pro35S:CYP81Q1* and *Pro35S:Steroleosin B/Pro35S:CYP81Q1* plants. Mean values ± standard variations were obtained from four technical replicates.

We previously showed that the heterologous expression of the sesamin synthase gene *CYP81Q1* in transgenic *Forsythia koreana* cell lines was sufficient for the conversion of endogenous pinoresinol to sesamin (Fig. 3a)^13–15^. Thus, in the present studies using *Arabidopsis* plants, we induced ectopic accumulation of sesamin by imposing a heterologous expression of the *CYP81Q1* gene. Specifically, the coding sequence of *CYP81Q1* was inserted downstream of the constitutively active cauliflower mosaic virus 35 S promoter (Pro35S), and the resulting construct was used to transform *Arabidopsis* plants. After confirming the heterologous expression of *CYP81Q1* in the transgenic *Pro35S:CYP81Q1 Arabidopsis* plants (Fig. 3b), we detected ectopic accumulation of sesamin in the shoots and roots of *Pro35S:CYP81Q1*, but not wild-type *Arabidopsis* plants (Fig. 3c,d).

**Figure 4:**
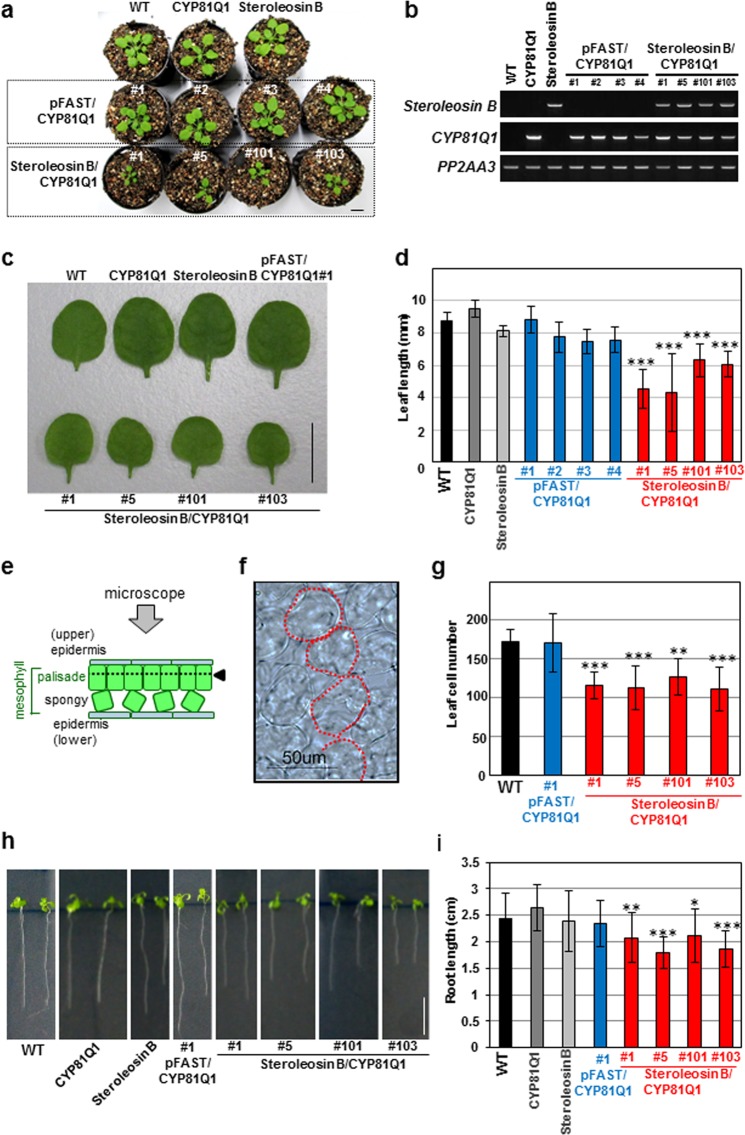
Ectopic expression of *Steroleosin B* and *CYP81Q1* genes in transgenic *Arabidopsis* plants suppress growth, leaf expansion and root elongation. (**a**) The rosettes of four-week-old wild-type and transgenic *Pro35S:CYP81Q1, Pro35S:Steroleosin B, pFAST/Pro35S:CYP81Q1*, and *Pro35S:Steroleosin B/Pro35S:CYP81Q1* plants. (**b**) Expression of *Steroleosin B* and *CYP81Q1* genes in wild-type, *Pro35S:CYP81Q1*, *Pro35S:Steroleosin B* and four T3 lines of *pFAST/Pro35S:CYP81Q1* and *Pro35S:Steroleosin B/Pro35S:CYP81Q1* plants. *PP2AA3* was used as an internal control. **(c)** Morphology of the forth leaves of wild-type and transgenic *Pro35S:CYP81Q1, Pro35S:Steroleosin B, pFAST/Pro35S:CYP81Q1*, and *Pro35S:Steroleosin B/Pro35S:CYP81Q1* plants. (**d**) The lengths of the fourth leaves of *Arabidopsis* plants (n = 8). **(e)** Model of a leaf cross-section indicating mesophyll palisade cells. The dotted lines marked by the arrowheads represent the focus plane of the microscopic observations in **(f)** and **(g)**. **(f)** Image of mesophyll palisade cells under microscopic observation. Red lines indicate the outlines of palisade cells. The mesophyll palisade cells form a simple round shape **(g)** Numbers of mesophyll palisade cells along the longitudinal axes of fourth leaves of *Arabidopsis* plants (n = 8). **(h)** Images of the wild-type and transgenic *Pro35S:CYP81Q1*, *Pro35S:Steroleosin B*, *pFAST/Pro35S:CYP81Q1* and *Pro35S:Steroleosin B/Pro35S:CYP81Q1* plants used for measurement of root length in **(g)**. **(g)** Lengths of main roots in *Arabidopsis* plants (n = 28–36). Bars = 1 cm in (**a**,**c**,**h**). Asterisks in **(d**,**g**,**i)** indicate significant differences relative to the wild-type (***P < 0.001. **P < 0.01, *P < 0.05). One-way ANOVA was performed, followed by Dunnett’s test. P < 0.05 was considered statistically significant. Error bars indicate standard deviations of indicated biological replicates.

Concurrently, the *Steroleosin B* gene was inserted downstream of the Pro35S sequence of the pFAST vector^16^ (*Pro35S:Steroleosin B*) and introduced into wild-type *Arabidopsis* plants. The resultant *Pro35S:Steroleosin B Arabidopsis* plants showed heterologous expression of the *Steroleosin B* gene (Supplementary Fig. S3a). Our phenotypic analysis demonstrated that *Pro35S:CYP81Q1* and *Pro35S:Steroleosin B* plants showed normal morphology and growth (Fig. 4a,c,d,g,i, Supplementary Fig. S3b). Therefore, ectopic accumulation of sesamin or Steroleosin B individually has no effect on physiology and morphology.

In further experiments, ectopic accumulation of both sesamin and Steroleosin B was achieved in *Arabidopsis* plants by introducing the *Pro35S:Steroleosin B* construct into *Pro35S:CYP81Q1 Arabidopsis* plants. The resulting 28 T1 generation lines of *Pro35S:Steroleosin B/Pro35S:CYP81Q1* plants were selected based on their herbicide resistance. *Pro35S:Steroleosin B/Pro35S:CYP81Q1 Arabidopsis* plants of the T1 generation were stunted and formed small leaves(Supplementary Fig. S4a). The six lines of *Pro35S:Steroleosin B/Pro35S:CYP81Q1 Arabidopsis* plants were significantly smaller than the vector control *pFAST/Pro35S:CYP81Q1 Arabidopsis* plants and showed defective leaf expansion (Supplementary Fig. S4a). The stunted plants died before fertilization or produced low numbers of viable seeds (Supplementary Fig. S4b). The remaining five *Pro35S:Steroleosin B/Pro35S:CYP81Q1 Arabidopsis* lines showed moderate defects in leaf expansion and produced seeds that were capable of germination.

To quantitatively and reproducibly compare morphological phenotypes, we established four homozygous T3 generation lines of *Pro35S:Steroleosin B/Pro35S:CYP81Q1* and *pFAST/Pro35S:CYP81Q1 Arabidopsis* plants (Fig. 4a). Initially, we confirmed the heterologous expression of *Steroleosin B* and *CYP81Q1* genes and ectopic accumulation of sesamin in the *Pro35S:Steroleosin B/Pro35S:CYP81Q1 Arabidopsis* plants (Figs 3d, 4b) and then performed quantitative analyses of leaf sizes (Fig. 4c,d). These measurements demonstrated that four-week-old *Pro35S:Steroleosin B/Pro35S:CYP81Q1 Arabidopsis* plants had significantly smaller leaves than the wild-type, *Pro35S:CYP81Q1*, *Pro35S:Steroleosin B*, and control *pFAST/Pro35S:CYP81Q1 Arabidopsis* plants (Fig. 4a,c,d). For comparison of cell number in leaves, the mesophyll palisade cells were counted (Fig. 4e,f). Notably, leaf size reduction was associated with a decreased number of cells in leaves (Fig. 4g). In addition, *Pro35S:Steroleosin B/Pro35S:CYP81Q1 Arabidopsis* plants showed moderately suppressed root elongation (Fig. 4h,i).

In contrast to *Pro35S:Steroleosin B/Pro35S:CYP81Q1 Arabidopsis* plants, single transgenic *Pro35S:CYP81Q1* and *Pro35S:Steroleosin B* plants exhibited normal morphologies (Fig. 4a,c,d and Supplementary Fig. S3b). These data confirm that ectopic accumulations of sesamin and Steroleosin B in *Arabidopsis* plants suppress leaf growth and root elongation, whereas accumulation of sesamin or Steroleosin B alone had no significant effect on morphology.

## Discussion

Steroleosins are minor integral proteins localized in the membrane of oil body in *S*. *indicum* and various other plant species^17–19^. Steroleosins exist as two isoforms, namely Steroleosin A and B, in *S. indicum*. They have an anchor domain in their N-terminal region that is required for the association with oil body membrane, whereas their C-terminal region has moderate sequence similarity to hydroxysteroid dehydrogenases (HSD) in animals^18,20^. HSDs in mammals have been shown to maintain steady-state concentrations of various steroid hormones by converting the hydroxy groups into ketones that are crucial for the biological activities of mammalian steroid hormones. No endogenous substrates for Steroleosins have been identified in plants, and thus the biological roles of Steroleosins have not been fully elucidated.

The present *in vitro* observations of sesamin binding to Steroleosin B (Figs 1 and 2) and consequent measurable growth suppression in transgenic *Arabidopsis* plants producing both sesamin and Steroleosin B (Fig. 4) offer insight into the biological roles of sesamin and its protein target. Our control plants, *Pro35S:CYP81Q1* and *Pro35S:Steroleosin B*, demonstrated that accumulation of sesamin or Steroleosin B alone caused no phenotypic changes, supportive of no interference between endogenous hydroxysterol dehydrogenases and sesamin or between Steroleosin B and endogenous lignans other than sesamin in transgenic *Arabidopsis* plants^21^. *Pro35S:Steroleosin B/Pro35S:CYP81Q1 Arabidopsis* plants showed measurable phenotypic changes indicative of their functional interactions *in planta*. These observations are quite valuable as no reliable, general methodology exists for detecting interactions between natural small molecules without chemical modification and their binding proteins *in planta*. Since expression of *CYP81Q1* and *Steroleosin B* genes was driven by the same Pro35S promoter, it is possible that sesamin and Steroleosin B accumulate in the same tissues and cells. *Pro35S:Steroleosin B/Pro35S:CYP81Q1 Arabidopsis* plants provide important clues for understanding the biological significance of sesamin and Steroleosin B.

Our data provide evidence that sesamin plays important roles in phenotypic expression profiles that are indispensable for the survival of plants in severe environments because growth suppression is a common and an important strategy for plants to cope with environmental changes, such as water availability and pathogen attack^22,23^. In particular, the suppression of leaf growth and root elongation by the combined presence of sesamin and Steroleosin B resembled that of transgenic *Arabidopsis* plants expressing an RNA interference gene for the *Hydroxysterol dehydrogenase 1* (*AtHSD1*)^24^. The amino acid sequence of the encoded AtHSD1 protein has a 56% similarity with that of Steroleosin B and the related enzyme activity plays roles in the regulation of leaf and root expansion and in seed germination^24^. Since AtHSD1 is involved in the brassinosteroid signalling pathway that regulates plant growth and development^24^, it is possible that sesamin and Steroleosin B potentially affect this endogenous pathway and influence the phenotype of *Pro35S:Steroleosin B/Pro35S:CYP81Q1 Arabidopsis* plants. Collectively, these observations suggest that accumulated sesamin and Steroleosin B interfere with endogenous AtHSD1 activity and suppress the growth of *Arabidopsis* plants. Although the putative activities of Steroleosin B and AtHSD1 are associated with dehydrogenases^24^, their endogenous substrates have not been identified. The elucidation of putative effects of sesamin binding to Steroleosin B, and of signalling cascade components that involve sesamin and Steroleosin B, is warranted to better understand the molecular basis of the biological function of Steroleosin B.

Sesamin primarily accumulates in seeds, and to a lesser extent in leaves, in sesame and its content varies among plant tissues and their developmental stages^5,25^. *CYP81Q1* and *Steroleosin B* genes are co-expressed during seed development and germination (Supplementary Fig. S2). In addition, transgenic *Pro35S:Steroleosin B/Pro35S:CYP81Q1 Arabidopsis* plants showed severe growth defects and produced less siliques compared to *Arabidopsis* plants harbouring *Pro35S:CYP81Q1* only (Supplementary Fig. S4b). From these data, we conclude that Steroleosin B may be required for sesamin to be biologically active and influence plant development; as a result, Steroleosin B and sesamin might be biologically relevant in developing seeds and germinating seedlings. The sesamin content of sesame leaves has been shown to be responsive to changes in light conditions^26^. Thus, sesamin in sesame plants could also be involved in various aspects of growth control to help cope with changes in environmental conditions.

Our approach using affinity nano-beads together with *in planta* evaluation of potential impact of sesamin–Steroleosin B interactions on *Arabidopsis* growth has provided new clues for understanding the biological significance of Steroleosin B-sesamin from the viewpoint of their physical interactions. Previous reports show considerable differences in sesamin content across various sesame cultivars^27,28^. However, correlation between phenotypic traits and sesamin content have not been previously described. The present data may suggest that Steroleosin B acts cooperatively with sesamin to control growth in sesame plants. Thus, more extensive surveillance of plant traits and measurements of accumulated Steroleosin B and sesamin levels in various sesame cultivars would be of great value to the understanding of their biological roles in sesame plants.

## Materials and Methods

### Synthesis of sesamin derivative

The experimental details are presented in the Supplementary Information.

### Preparation of sesame seedling extracts

In order to obtain extracts, 1 g samples of fresh immature seedlings were harvested and ground into fine powder under liquid nitrogen using a mortar and pestle. Each powdered sample was dissolved in 2.5 ml of extraction buffer containing 100-mM Tris-Cl (pH 8.0), 0.1% β-mercaptoethanol and a cocktail of protease inhibitors (1× Complete EDTA-free, Roche, Japan). Homogenates were then passed twice through nylon mesh and desalted twice using a PD-10 column (GE Healthcare, Japan). Finally, desalted homogenates were subjected to nano-bead-based affinity screening for SBPs.

### Preparation of SIB and AIB

First, 2 mg aliquots of magnetic FG-nano-beads (Tamagawa Seiki Co.,Ltd.) were treated with sesamin probe **4** at low and high concentrations of 0.2 and 2 mM, respectively, in the presence of N-hydroxysuccinimide (10 mM) and 1-ethyl-3-(3-dimethylaminopropyl)-carbodiimide (10 mM) in dimethylformamide (DMF) at room temperature for 12 h. After washing with DMF, unreacted amino groups on nano-beads were masked using acetic anhydride (200 mM) in DMF for 2 h to obtain SIB. Next, SIB were washed sequentially with DMF, MeOH and phosphate buffered saline (PBS) and subjected to affinity pull-down assays. AIB were prepared by acetylation of amine beads, as described above, without sesamin probe **4** treatment. AIB were used as the control beads.

### Affinity purification of SBP using SIB

To purify SBPs, 2 mg samples of SIB were pre-equilibrated with PBS and incubated with the aforementioned sesame seedling extracts in 1 ml aliquots of PBS containing 0.1% NP-40; sesamin was used as a competitor at 2 mM. After washing with PBS, bound proteins were eluted with SDS containing PBS at 90 °C for 5 min, separated using SDS-PAGE and visualised by silver staining. The excised bands were then subjected to in-gel digestion with trypsin. Peptide mass fingerprint analyses were performed using nano liquid chromatography-mass spectrometry (nano LC-MS; Thermo Easy nLC1000 equipped Thermo Orbitrap Elite) and analysed by Mascot search software.

### Production of GST-Steroleosin B fusion proteins

Cells from *Escherichia coli* strain Rosetta2 (DE3; Merck Millipore, Japan) were transformed with pDEST15-GST-Steroleosin B and pre-cultured overnight in 3-ml of LB medium at 37 °C. Flasks containing 50 ml of LB medium supplemented with 50-µg/ml kanamycin were inoculated with 500 µl aliquots of saturated culture. Cells were cultivated until the optical density at 600 nm (OD_600_) reached 1.0. The cultures were then supplemented with 1 mM isopropyl β-D-1-thiogalactopyranoside (IPTG) and cultured for a further 3 h at 23 °C. Next, the cells were harvested by centrifugation at 10,000 × *g* for 10 min at 4 °C. After removal of supernatant, pellets were stored at −80 °C until purification of GST-fused Steroleosin B, as described elsewhere. Briefly, cell pellets were re-suspended in 15 ml aliquots of ice cold extraction buffer containing 1× PBS, 0.1% β-mercaptoethanol and 1× Complete EDTA-free (Sigma-Aldrich, Japan) and then ultrasonically lysed on ice. Homogenates were finally centrifuged at 10,000 × *g* for 15 min at 4 °C before being subjected to the pull-down assays using SIB, as described above.

### STD-NMR analyses of sesamin and GST-fused Steroleosin B

STD-NMR spectra were acquired using a Bruker AVANCE III HD 800 spectrometer equipped with a 5 mm TCI cryogenic probe and a Z-axis gradient (Bruker Biospin AG, Switzerland). All spectra were measured at 298 K using Wilmad 5 mm NMR tubes. Standard Bruker pulse sequences were employed and residual water signals were suppressed by applying an excitation sculpting sequence.

To prepare the samples for the NMR experiments, sesamin (60 μM) and GST-Steroleosin B or control GST (0.5 μM) were dissolved in 500 μl of PBS that had been prepared with deuterium oxide containing 5% dimethyl sulfoxide-*d*_6_ (DMSO-*d*_6_). On-resonance irradiation of proteins was performed with a chemical shift of 1 part per million (ppm) and off-resonance irradiation was applied at 30 ppm. Selective irradiation was achieved using Gaussian pulses of 50 ms, resulting in a total saturation time of 3.0 s. Other acquisition parameters were as follows: number of data points, 32 K; spectral width, 9615 Hz; relaxation delay, 5.0 s; number of scans, 512 and receiver gain, 203. Chemical shifts are reported relative to peaks of DMSO-*d*_6_, δ_H_ observed at 2.50 ppm.

### Plant materials and growth conditions

*S. indicum* plants were grown outdoors in pots unless otherwise indicated, and developing seeds were harvested during designated developmental stages^5^. *Arabidopsis thaliana* ecotype Col-0 plants were grown in soil or on plates containing half-strength MS salts, 0.5 g/l MES and 5 g/l sucrose at 22 °C under a 16 h light/8 h dark photocycle. In preparation for harvesting seeds, *Arabidopsis* plants of various genotypes were arranged side by side and grown on soil until senescence after maturity and then air-dried. In preparation for transforming the *Arabidopsis* plants, *Agrobacterium tumefaciens* GV3101 was transformed using the indicated plasmids (as below) using electroporation. The resulting bacteria were used to transform plants using the floral-dip method^29^ with minor modifications. Transgenic *Arabidopsis* plants were then selected on plates containing appropriate antibiotics or herbicides. Prior to the determination of sesamin content, the *Arabidopsis* plants were grown vertically for three weeks on plates.

### Construction of plasmids

To clone the coding sequence of the *Steroleosin B* gene (accession number AAM46847), cDNA was generated using total RNA from developing sesame seeds with oligo dT primers and SuperScript III (ThermoFisher Scientific, Japan) and amplified using the appropriate primer sets. To establish protein production in *E. coli*, the coding sequence of *Steroleosin B* was subcloned into the vector pDEST15 (pDEST15-GST-Steroleosin B; ThermoFisher Scientific). *Arabidopsis* plants were transformed by inserting the coding sequences of *CYP81Q1* (accession number AB194714) and *Steroleosin B* genes downstream of the Pro35S promoter using pBINplus^30^ and pFAST^14^ plasmids, respectively.

### Analysis of gene expression

Total RNA was isolated from individual tissues of sesame and *Arabidopsis* plants using RNeasy Plant Mini Kits (Qiagen) and reverse transcribed using SuperScript III (Life Technologies) using oligo dT primers. Aliquots of cDNAs were amplified using ExTaq (Takara, Japan) with the primer sets listed in Supplementary Table S3 and a conventional thermal cycler (Veriti 200; Applied Biosystems). The amplified fragments were then separated on agarose gels and visualized using ethidium bromide. Quantitative real-time PCR analyses were performed using a StepOnePlus real-time PCR system and SYBR Green PCR Master Mix Kits (Life Technologies) with the appropriate primer sets (Supplementary Table S3). *CYP81Q1* gene expression levels were quantified relative to *ACT2* expression using StepOne Software v2.0 (Life Technologies).

### Phenotype analyses of *Arabidopsis* plants

To quantify leaf length, the leaves at the fourth position of four-week-old *Arabidopsis* plants were photographed and measured along the longitudinal axis. Comparisons of leaf cell numbers were made by reducing the transparency of the leaf at the fourth position^31^ and counting the mesophyll palisade cells in the sub-epidermal layers along the longitudinal axis using a light microscope (ECLIPSE; Nikon). To quantify root length, the main roots of two-week-old seedlings were detached and directly measured using a ruler. Statistical analyses were performed using BellCurve for Excel software with the default settings (https://bellcurve.jp/ex/). One-way ANOVA was performed, followed by Dunnett’s test. *P* < 0.05 was considered statistically significant.

### Measurement of sesamin content in *Arabidopsis* plants

*Arabidopsis* plants were freeze-dried using a FDU-2110 device (EYELA), extracted twice in 50% methanol at 65 °C for 1 h and then centrifuged at 20,000 × *g* for 3 min. The resulting supernatants were concentrated under vacuum and filtered using a Millex-LH column (Millipore). To determine the sesamin content, filtrates were applied to an ACQUITY UPLC system (Waters Corp.), as described previously with several modifications^32^. Briefly, 3-μl aliquots of filtrates were added to ACQUITY UPLC HSS C18 Columns (2.1 × 150 mm, 1.8 μm) at 40 °C and analysed with a Waters 470 Scanning Fluorescence Detector (Waters Corp.) with excitation and emission wavelengths set at 280 and 340 nm, respectively. Mobile phases comprised 5% (v/v) acetonitrile in ultra pure water containing 0.05% (v/v) acetic acid (solvent A) and 95% (v/v) acetonitrile in ultra pure water with 0.05% (v/v) acetic acid (solvent B). The gradient elution programme was as follows: 60–90% B for 0–6 min, 90–95% B for 6–6.5 min, 95–90% B for 6.5–8 min, 90–60% B for 8–12 min and 60% B for 12–13 min at a flow rate of 0.3 ml/min. Chromatographic peaks were identified in comparison with the retention times of authentic sesamin and quantified from the peak areas using an external standard method. The resulting chromatograms were then analysed using Waters Empower 2 software.

## Supplementary information

Supplementary information
