# Author Correction: Identification of a binding protein for sesamin and characterization of its roles in plant growth

**DOI:** 10.1038/s41598-020-64237-4

**Published:** 2020-05-07

**Authors:** Masayuki Tera, Tomotsugu Koyama, Jun Murata, Ayako Furukawa, Shoko Mori, Toshiaki Azuma, Takehiro Watanabe, Katsuhito Hori, Atsushi Okazawa, Yasuaki Kabe, Makoto Suematsu, Honoo Satake, Eiichiro Ono, Manabu Horikawa

**Affiliations:** 1 Bioorganic Research Institute, Suntory Foundation for Life Sciences (SUNBOR), 8-1-1 Seikadai, Seika, Soraku, Kyoto, 619-0284 Japan; 2 Graduate School of Engineering, Osaka University, 2-1 Yamadaoka, Suita, Osaka 565-0871 Japan; 3 Graduate School of Life and Environmental Sciences, Osaka Prefecture University, 1-1 Gakuen-cho, Naka-ku, Sakai, Osaka 599-8531 Japan; 4 Department of Biochemistry, Keio University School of Medicine, 35 Shinanomachi, Shinjyuku-ku, Tokyo 160-8582 Japan; 5 Japan Agency for Medical Research and Development (AMED), Core Research for Evolutional Science and Technology (CREST), 1-7-1 Otemachi, Chiyoda-ku, 100-0004 Japan; 6 Research Institute, Suntory Global Innovation Center Ltd (SIC), 8-1-1 Seikadai, Seika, Soraku, Kyoto 619-0284 Japan

Correction to: *Scientific Reports* 10.1038/s41598-019-45003-7, published online 14 June 2019

The Supplementary Information file originally published with this Article contained errors.

In the ‘Detailed experimental procedures:’ section,

“106.57 (c-2’)”

now reads:

“10657 (C-2’)”

and

“mp 91–92 °C”

now reads:

“m.p. mp 91–92 °C”.

Finally, the original file contained tracked changes.

These errors have been corrected in the Supplementary Information that now accompanies the Article.

