# Supplementary material for: Identification of a binding protein for sesamin and characterization of its roles in plant growth
Source: Sci Rep. 2019 Jun 14;9:8631. doi: 10.1038/s41598-019-45003-7 (PMC6570762; doi:10.1038/s41598-019-45003-7)
Supplement: Supplementary file 1 — Supplementary information [file 41598_2019_45003_MOESM1_ESM.docx]

**Supplementary Information**

**Identification of a binding protein for sesamin and characterization of its roles in plant growth**

**Authors:** Masayuki Tera^1†^, Tomotsugu Koyama^1†^, Jun Murata^1^, Ayako Furukawa^1^, Shoko Mori^1^, Toshiaki Azuma^1^, Takehiro Watanabe^1^, Katsuhito Hori^2^, Atsushi Okazawa^2,3^, Yasuaki Kabe^4,5^, Makoto Suematsu^4^, Honoo Satake^1^, Eiichiro Ono^6^ and Manabu Horikawa^1*^

**Affiliations:**

^1^Bioorganic Research Institute, Suntory Foundation for Life Sciences (SUNBOR), 8-1-1 Seikadai, Seika, Soraku, Kyoto 619-0284, Japan.

^2^Graduate School of Engineering, Osaka University, 2-1 Yamadaoka, Suita, Osaka 565-0871, Japan

^3^Graduate School of Life and Environmental Sciences, Osaka Prefecture University, 1-1 Gakuen-cho, Naka-ku, Sakai, Osaka 599-8531, Japan

^4^Department of Biochemistry, Keio University School of Medicine, 35 Shinanomachi,

Shinjyuku-ku, Tokyo, 160-8582, Japan

^5^Japan Agency for Medical Research and Development (AMED), Core Research for Evolutional Science and Technology (CREST), 1-7-1, Otemachi, Chiyoda-ku, 100-1004, Japan.

^6^Research Institute, Suntory Global Innovation Center Ltd (SIC), 8-1-1 Seikadai, Seika, Soraku, Kyoto 619-0284, Japan.

**Synthesis of sesamin derivatives**

**General information:**

^1^H NMR spectra were recorded on a Bruker AVANCE III HD 400 or 800 MHz spectrometer at ambient temperature with CDCl_3_ as the solvent unless otherwise stated. Chemical shifts are reported in parts per million relative to chloroform-*d* (^1^H, δ 7.26; ^13^C, δ 77.00) or acetone-*d_6_* (^1^H, δ 2.05; ^13^C, δ 29.92). Data for ^1^H NMR are reported as follows: chemical shift, multiplicity (s = singlet, d = doublet, t = triplet, q = quartet, m = multiplet), integration and coupling constants. High-resolution mass spectra were obtained using an ion-trap time-of-flight mass spectrometer (Shimadzu LCMS-IT-TOF). Optical rotations were recorded on a JASCO DIP-1000 digital polarimeter at 589 nm and are recorded as [α]_D_ (concentration in g/100 ml solvent). Flash chromatography was performed using 200-400 mesh silica gel. Yields refer to chromatographically and spectroscopically pure materials, unless otherwise stated. All reagents were used as supplied by Nacalai tesque, TCI, and Sigma-Aldrich. Sesamin was purchased from ChromaDex, Inc.. Magnetic nano beads with amine linkers were purchased from Tamagawa Seiki (Nagano, Japan).

**Detailed experimental procedures:**

Synthesis of 4-((1S,3aR,4S,6aR)-4-(benzo[d][1,3]dioxol-5-yl)hexahydrofuro[3,4-c]furan-1-yl)-　2-methoxyphenol (**2**)





Sesamin (3.0 g, 8.48 mmol) and diisobutylaluminum hydride (^i^Bu_2_AlH, 25 wt. % in toluene, density: 0.846 g/ml, 22.8 ml, 33.9 mmol) dissolved in toluene (60 ml) were refluxed for 1 h. The reaction mixture was cooled to room temperature then quenched by crushed ice and aqueous HCl solution (1 M) carefully and extracted three times with EtOAc. The combined organic layers were washed with brine, dried over anhydrous MgSO_4_ and concentrated *in vacuo*. The residue was purified by flash chromatography on silica gel with n-hexane-EtOAc (v/v = 4:1) to afford a mixture of **1** and **2**. Then a complete separation of **1** and **2** was performed by chiral column chromatography (DAICEL chiralcell OB-H, 20 mm I.D. × 250 mm) with n-hexane-2-propanol (v/v = 9:1) to obtain **1** (435 mg, 1.23 mmol, 14.5 %) and **2** (417 mg, 1.17 mmol, 13.7 %), respectively.

**1**: mp 100–102 °C; [*α*] +57.2° (c 1.1, CHCl_3_); ^1^H NMR (800 MHz, CDCl_3_) *δ* 3.03 (1H, *m*, H-8'), 3.09 (1H, *m*, H-8), 3.87 (2H, *m*, H-9, 9'), 3.88 (3H, *s*, OCH_3_), 4.22 (1H, *dd*, J = 7.0, 9.2 Hz, H-9), 4.25 (1H, *dd*, J = 7.2, 9.1 Hz, H-9'), 4.70 (1H, *d*, J = 5.4 Hz, H-7'), 4.73 (1H, *d*, J = 5.0 Hz, H-7), 5.63 (1H, *s*, OH), 5.95 (2H, *s*, OCH_2_O), 6.78 (1H, *d*, J = 8.0 Hz, H-5'), 6.80 (1H, *dd*, J = 1.4, 8.0 Hz, H-6'), 6.82 (1H, *d*, J = 8.2 Hz, H-5), 6.84 (1H, *dd*, J = 1.6, 8.2 Hz, H-6), 6.85 (1H, *d*, J = 1.4 Hz, H-2'), 6.92 (1H, *d*, J = 1.6 Hz, H-2); ^13^C NMR (200 MHz, CDCl_3_) *δ* 54.1 (C-8), 54.4 (C-8'), 56.0 (OCH_3_), 71.5 (C-9), 71.9 (C-9'), 85.5 (C-7), 85.9 (C-7'), 101.0 (OCH_2_O), 106.5 (C-2’), 108.2 (C-5'), 110.5 (C-5), 112.3 (C-2), 117.6 (C-6), 119.4 (C-6'), 134.4 (C-1), 135.1 (C-1'), 145.7 (C-3), 146.1 (C-4), 147.1 (C-4'), 147.9 (C-3'). HRMS (ESI): [M+Na]^+^ calculated for C_20_H_20_O_6_Na: m/z 377.1234, found: 337.1245.

The ^1^H and ^13^C NMR signals (Supplementary Fig. S5 and S6) of **1** were assigned by HSQC, HMBC and NOESY experiments (Supplementary Fig. S7, S8 and S9). The result of HMBC experiment of **1** shows Supplementary Table S4.

**2**: mp 61–62 °C; [*α*] +68.5 ° (*c* 1.4, CHCl_3_); ^1^H NMR (800 MHz, CDCl_3_) *δ* 3.06 (1H, *m*, H-8'), 3.08 (1H, *m*, H-8), 3.87 (1H, *dd*, *J* = 3.9, 9.0 Hz, H-9'), 3.88 (1H, *dd*, *J* = 3.9, 9.0 Hz, H-9), 3.90 (3H, *s*, OCH_3_), 4.23 (1H, *dd*, *J* = 6.5, 9.6 Hz, H-9), 4.25 (1H, *dd*, *J* = 6.9, 9.8 Hz, H-9'), 4.72 (1H, *d*, *J* = 4.6 Hz, H-7), 4.73, (1H, *d*, *J* = 4.3 Hz, H-7'), 5.62 (1H, *s*, OH), 5.95 (2H, *s*, OCH_2_O), 6.78 (1H, *d*, *J* = 7.9 Hz, H-5'), 6.81 (1H, *dd*, *J* = 1.0, 7.9 Hz, H-6'), 6.82 (1H, *dd*, *J* = 1.5, 7.8 Hz, H-6), 6.85 (1H, *d*, *J* = 1.0Hz, H-2’), 6.89 (1H, *d*, *J* = 1.5 Hz, H-2), 6.89 (1H, *d*, *J* = 7.8 Hz, H-5); ^13^C NMR (200 MHz, CDCl_3_) *δ* 54.1 (C-8), 54.3 (C-8'), 55.9 (OCH_3_), 71.6 (C-9), 71.7 (C-9'), 85.78 (C-7), 85.83 (C-7'), 101.0 (OCH_2_O), 10657 (C-2'), 108.2 (C-5'), 108.6 (C-2), 114.2 (C-5), 119.0 (C-6), 119.3 (C-6'), 132.8 (C-1), 135.1 (C-1'), 145.2 (C-4), 146.7 (C-3), 147.1 (C-4'), 147.9 (C-3'). HRMS (ESI): [M+Na]^+^ calculated for C_20_H_20_O_6_Na: m/z 377.1234, found: 337.1245.

The ^1^H and ^13^C NMR signals (Supplementary Fig. S10 and S11) of **2** were assigned by HSQC, HMBC and NOESY experiments (Supplementary Fig. S12, S13 and S14). The result of HMBC experiment of **2** is shown in Supplementary Table S5.

Synthesis of 2-(4-((1S,3aR,4S,6aR)-4-(benzo[d][1,3]dioxol-5-yl)hexahydrofuro[3,4-c]furan-1-　yl)-2-methoxyphenoxy)acetic acid (**4**)





　Piperitol (**2**) (100 mg, 0.28 mmol) and methyl bromoacetate (38.8 µl, 0.42 mmol) dissolved in DMF were suspended K_2_CO_3_ (58.2 mg, 0.42 mmol) and then vigorously stirred at room temperature for 2 h. The reaction mixture was quenched with 10% citric acid aqueous solution at 0°C and then extracted twice with EtOAc. The combined organic layers were washed twice with H_2_O and brine, dried over anhydrous MgSO_4_ and concentrated *in vacuo*. The residue was purified by flash chromatography on silica gel with n-hexane-EtOAc (v/v = 4:1) to afford an ester **3**. Hydrolysis of **3** with 1N NaOH aqueous solution (2 ml) in tetrahydrofuran (2 ml) at room temperature for 2 h was followed by the reverse phase HPLC preparation to provide the carboxylate **4** (69 mg, 0.17 mmol, 59%, 2 steps).

**3**: m.p. mp 91–92 °C; [*α*] +54.4 ° (*c* 1.1, CHCl_3_); ^1^H NMR (400 MHz, CDCl_3_) *δ* 3.07 (2H, *m*, H-8, 8'), 3.79 (3H, *s*, COCH_3_), 3.875, 3.879 (each 1H, *dd*, *J* = 3.9, 9.2 Hz, H-9, 9'), 3.90 (3H, *s*, OCH_3_), 4.238, 4.244 (each 1H, *dd*, *J* = 6.7, 9.2 Hz, H-9, 9'), 4.69 (2H, *s*, OCH_2_CO), 4.72, 4.74 (each 1H, *d*, *J* = 4.9 Hz, H-7, 7'), 5.95 (2H, *s*, OCH_2_O), 6.77 (1H, *d*, *J* = 7.9 Hz, H-5'), 6.79 (1H, *d*, *J* = 8.3 Hz, H-5), 6.80 (1H, *dd*, *J* = 1.3, 7.9 Hz, H-6'), 6.83 (1H, *dd*, *J* = 1.6, 8.3 Hz, H-6), 6.85 (1H, *d*, *J* = 1.3 Hz, H-2'), 6.93 (1H, *d*, *J* = 1.6 Hz, H-2); ^13^C NMR (100 MHz, CDCl_3_) *δ* 52.2 (CO*C*H_3_), 54.2, 54.3 (C-8, 8'), 56.0 (OCH_3_), 66.5 (O*C*H_2_CO), 71.7, 71.8 (C-9, 9'), 85.6, 85.8 (C-7, 7'), 101.0 (OCH_2_O), 106.5 (C-2'), 108.2 (C-5'), 109.8 (C-2), 114.1 (C-5), 118.1 (C-6), 119.3 (C-6'), 135.0 (C-1'), 135.4 (C-1), 146.7 (C-4), 147.1 (C-4'), 147.9 (C-3'), 149.8 (C-3), 169.4 (CO). HRMS (ESI): [M+Na]^+^ calculated for C_23_H_24_O_8_Na: m/z 451.4211, found: 451.4207.

The ^1^H and ^13^C NMR signals (Supplementary Fig. S15 and S16) of **3** were assigned by HSQC, HMBC and NOESY experiments (Supplementary Fig. S17, S18 and S19). The result of HMBC experiment of **3** is shown in Supplementary Table S6.

**4**: mp 146–148 °C; [*α*] +51.2 ° (*c* 1.0, acetone); ^1^H NMR (400 MHz, acetone-*d*_6_) *δ* 3.08 (2H, *m*, H-8, 8'), 3.83 (1H, *dd*, *J* = 4.4, 9.1 Hz, H-9'), 3.84 (1H, *dd*, *J* = 4.1, 9.0 Hz, H-9), 3.85 (3H, *s*, OCH_3_), 4.215 (1H, *dd*, *J* = 6.8, 9.1 Hz, H-9'), 4.219 (1H, *dd*, *J* = 6.9, 9.0 Hz, H-9), 4.69 (2H, *s*, OCH_2_CO), 4.69 (1H, *d*, *J* = 4.9 Hz, H-7'), 4.71 (1H, *d*, *J* = 4.9 Hz, H-7), 5.97 (2H, *s*, OCH_2_O), 6.80 (1H, *d*, *J* = 8.0 Hz, H-5'), 6.87 (1H, H-6'), 6.89 (1H, H-6), 6.90 (1H, H-2'), 6.92 (1H, *d*, *J* = 8.3 Hz, H-5), 7.04 (1H, *d*, *J* = 0.9 Hz, H-2); ^13^C NMR (100 MHz, acetone-*d*_6_) *δ* 55.3, 55.5 (C-8, 8'), 56.3 (OCH_3_), 66.7 (O*C*H_2_CO), 72.3, 72.4 (C-9, 9'), 86.4, 86.6 (C-7, 7'), 102.0 (OCH_2_O), 107.4 (C-2'), 108.8 (C-5'), 111.5 (C-2), 115.4 (C-5), 119.1 (C-6), 120.2 (C-6'), 136.9 (C-1'), 137.0 (C-1), 147.9 (C-4), 148.0 (C-3'), 148.9 (C-4'), 150.8 (C-3), 170.6 (CO). HRMS (ESI): [M+Na]^+^ calculated for C_22_H_22_O_8_Na: m/z 437.3945, found: 437.3942.

The ^1^H and ^13^C NMR signals (Supplementary Fig. S20 and S21) of **4** were assigned by HSQC, HMBC and NOESY experiments (Supplementary Fig. S22, S23 and S24). The result of HMBC experiment of **4** is shown in Supplementary Table S7.

**Supplementary Table S1. Result of Mascot analysis**

Database : NCBInr

Organism species : all entries

Threshold level for the total score　: >45 (shown the listed data over 90)

**Supplementary Table S2. List of identified peptide sequences for the emzymatic digestion of the SDS target band by LC-MS/MS.**

**Supplementary Table S3. Primers used in this study.**


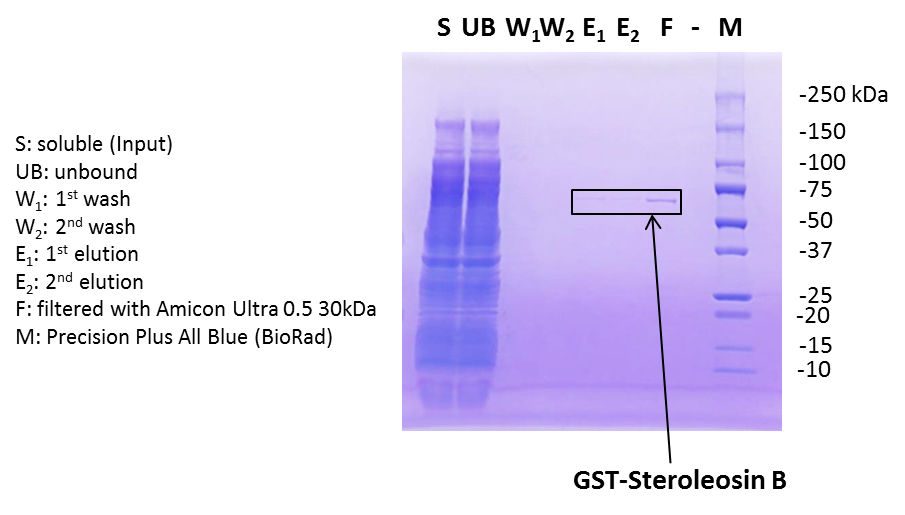


**Supplementary Figure S1. Production and purification of GST-Steroleosin B fusion protein for STD-NMR.** *E. coli* strain Rosetta2 (DE3) (Merck Millipore, Japan) was transformed with pDEST15-GST-Steroleosin B and pre-cultured overnight in 3 ml of LB medium at 37°C. A total of 10 flasks containing 50 ml of LB medium supplemented with 50 µg/ml of kanamycin was inoculated with 500 µl each of the saturated overnight culture, and further cultured until OD600 reached 1.0. The cultures were transferred to 23°C, supplemented with 1 mM IPTG and further cultured for 3 h. The cells were harvested by centrifugation at 10,000 × *g* for 10 min at 4°C and stored at −80°C until protein purification. GST-Steroleosin B was purified as described elsewhere. Briefly, the pellet from each of 50 ml culture was combined and re-suspended in 15 ml of ice cold Extraction buffer [1 × PBS, 0.1% β-mercaptoethanol, 1 × Complete EDTA-free (Sigma-Aldrich, Japan)] and lysed by ultrasonic on ice. The homogenate was centrifuged at 10,000 × *g* for 15 min at 4°C. The supernatant was incubated batch-wise with 200 µl bed volume of Glutathione Sepharose 4B (GE Healthcare, Japan) that was pre-equilibrated with Extraction buffer with gentle rotation for 2 h at 4°C. The resin was washed three times by centrifugation at 1,000 × *g* for 1 min at 4°C, followed by the removal of the supernatant and incubation for 10 min at 4°C with 20 times volume of Extraction buffer. For protein elution, the resin was incubated with 2 ml of Extraction buffer containing 5 mM reduced glutathione for 1 h at 4°C with gentle rotation, and centrifuged at 1,000 × *g* for 10 min at 4°C. The buffer of the recombinant protein was replaced to 1 × PBS without β-mercaptoethanol and Complete EDTA-free using Amicon Ultra 0.5 (30K) (Merck Millipore) according to the manufacturer’s instruction. For STD-NMR experiments, the buffer was replaced to 1 × PBS prepared with deuterated distilled water using Amicon Ultra 0.5 (30K).


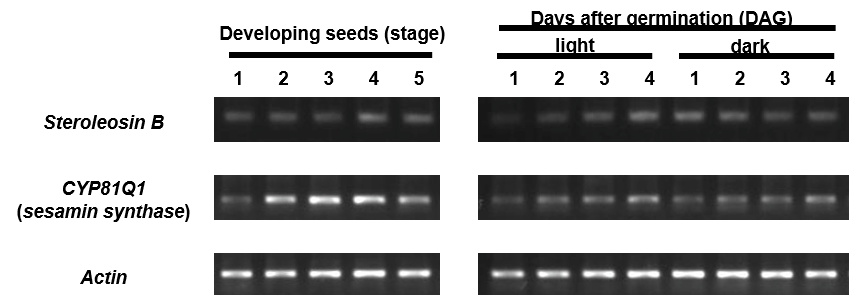


**Supplementary Figure S2.** ***Steroleosin B* is co-expressed with *CYP81Q1* throughout the seed development and post-germination stages.** Total RNA was extracted from developing sesame seeds (stages 1–5 according to Ono *et al.* 2006^5^) as well as germinating sesame seeds either under long day photo period (light) or continuous dark (dark) condition, and was subjected to RT-PCR analysis using primer sets for *Steroleosin B* and *CYP81Q1.* The expression of *Actin* was monitored as an internal control. The results indicate that the expression of *Steroleosin B* coincides with that of *CYP81Q1* during most of the stages of seed development as well as germination stages in sesame plants. DAG; days after germination.

**Supplementary Figure S3. *Pro35S:Steroleosin B Arabidopsis* plants of T1 generation.**

(**a**) Expression of *Steroleosin B* gene in *Pro35S:Steroleosin B Arabidopsis* plants. Aliquots of total RNA were individually prepared from herbicide-resistant plants of T1 generation and subjected to RT-PCR. *PP2AA3* was served as an internal control.

(**b**) Rosettes of wild-type and transgenic *Pro35S:Steroleosin B* plants. Bar = 1 cm.

**Supplementary Figure S4. *Pro35S:Steroleosin B/Pro35S:CYP81Q1* and *pFAST/Pro35S:CYP81Q1 Arabidopsis* plants of T1 generation.**

Severe phenotypes in leaves (**a**) and senescent plants (**b**) following ectopic expression of *Steroleosin B* and *CYP81Q1* genes in the *Arabidopsis* plants of the T1 generation; pFAST, vector control; arrows in (**b**) indicate the apexes of respective plants. Bar = 1 cm.


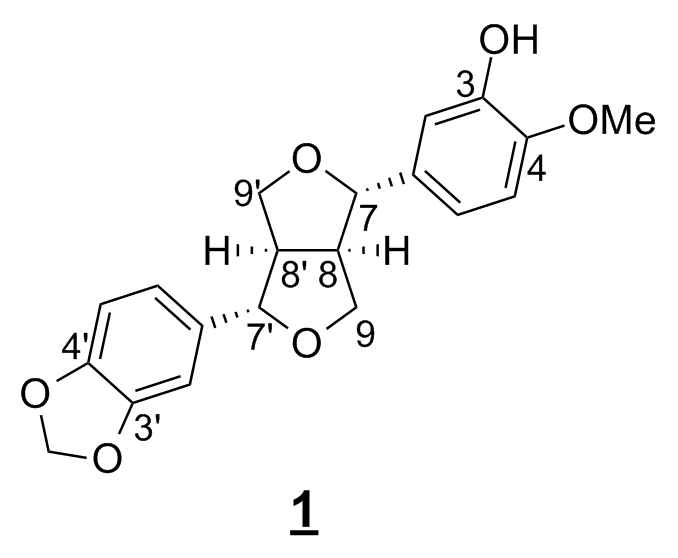

**Supplementary Figure S5.** The ^1^H NMR (CDCl_3_) spectrum of **1**.

**Supplementary Figure S6.** The ^13^C NMR (CDCl_3_) spectrum of **1**.

**Supplementary Figure S7.** The HSQC spectrum of **1.**

**Supplementary Figure S8.** The HMBC spectrum of **1**.


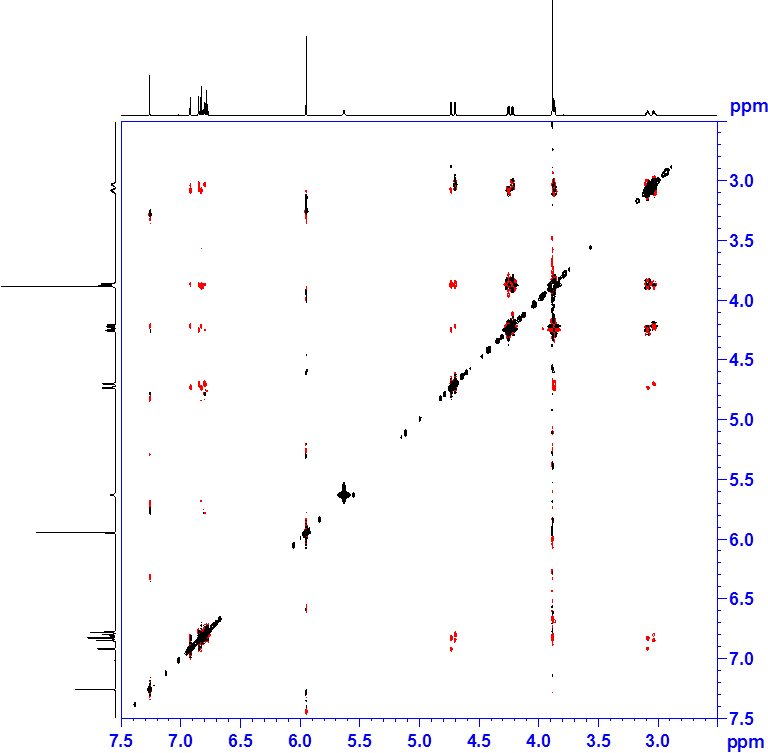


**Supplementary Figure S9.** The NOESY spectrum of **1**.

| Position | HMBC | Position | HMBC |
| --- | --- | --- | --- |
| 2 | 1, 3, 4, 6,7 | 2' | 1', 3', 4', 6', 7' |
| 5 | 1, 3, 4, 6 | 5' | 1', 3', 4' |
| 6 | 1, 2, 4, 7 | 6' | 1', 2', 4', 7' |
| 7 | 1, 2, 6, 8, 9, 8', 9' | 7' | 8, 9, 1', 2', 6', 8', 9' |
| 8 | 1, 9, 7', 8' | 8' | 7, 8, 1', 9' |
| 9 | 7, 8, 7', 8' | 9' | 7, 8, 7', 8' |
| OH | 2, 3, 4 | OCH_2_O | 3', 4' |
| OCH_3_ | 4 |  |  |

**Supplementary Table S4.** HMBC data of **1**.


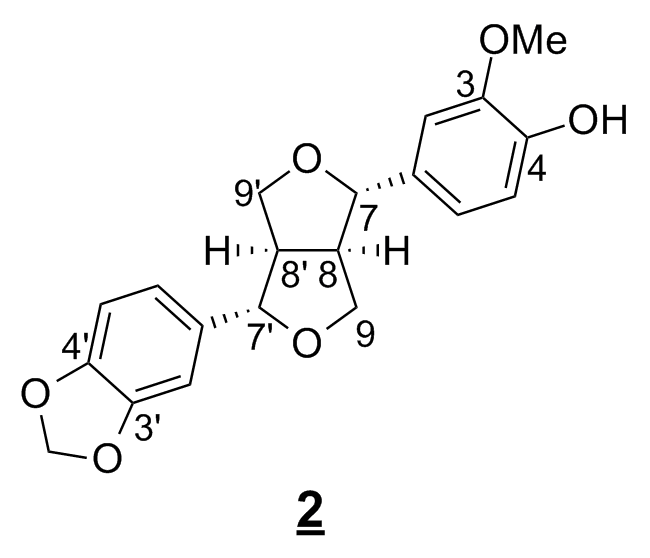

**Supplementary Figure S10.** The ^1^H NMR (CDCl_3_) spectrum of **2**.

**Supplementary Figure S11.** The ^13^C NMR (CDCl_3_) spectrum of **2**.

**Supplementary Figure S12.** The HSQC spectrum of **2**.

**Supplementary Figure S13.** The HMBC spectrum of **2**.


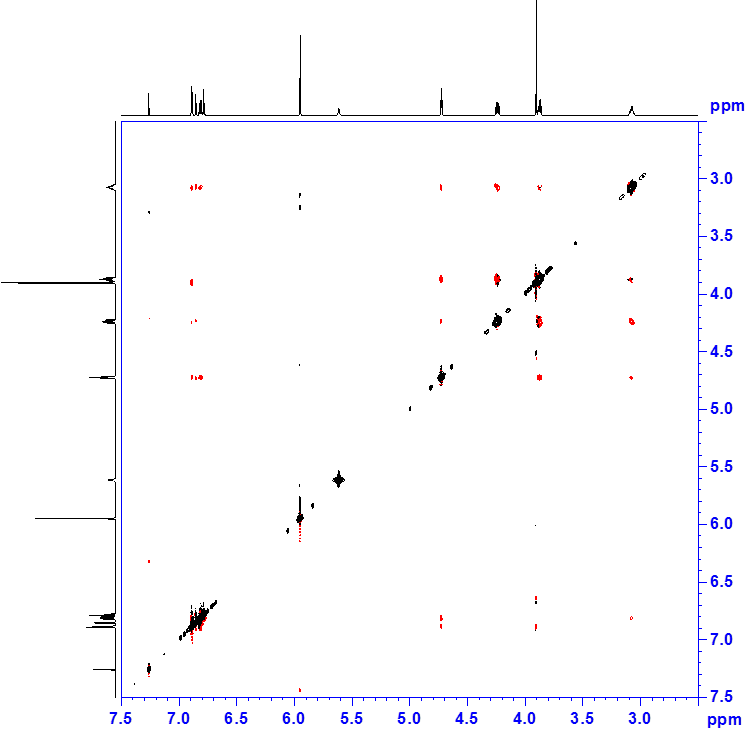


**Supplementary Figure S14.** The NOESY spectrum of **2**.

**Supplementary Table S5.** HMBC data of **2**.

| Position | HMBC | Position | HMBC |
| --- | --- | --- | --- |
| 2 | 1, 3, 4, 6,7 | 2' | 1', 3', 4', 6', 7' |
| 5 | 1, 3, 4, 6 | 5' | 1', 3', 4' |
| 6 | 2, 4, 5, 7 | 6' | 1', 2', 4', 7' |
| 7 | 1, 2, 6, 8, 9, 8', 9' | 7' | 8, 9, 1', 2', 6', 8', 9' |
| 8 | 1, 7, 9, 7', 8', 9' | 8' | 7, 8, 9, 1', 7', 9' |
| 9 | 7, 8, 7', 8' | 9' | 7, 8, 7', 8' |
| OH | 3, 4, 5 | OCH_2_O | 3', 4' |
| OCH_3_ | 3 |  |  |


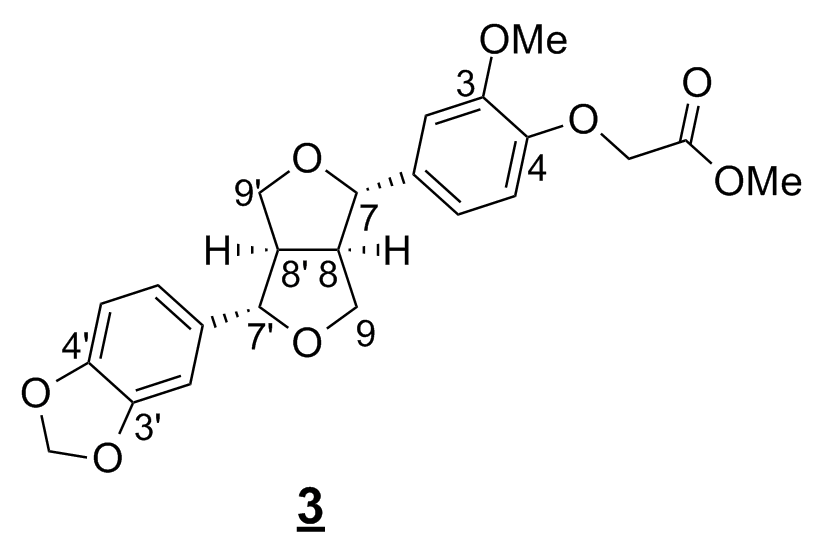

**Supplementary Figure S15.** The ^1^H NMR (CDCl_3_) spectrum of **3**.

**Supplementary Figure S16.** The ^13^C NMR (CDCl_3_) spectrum of **3**.

**Supplementary Figure S17.** The HSQC spectrum of **3**.

**Supplementary Figure S18.** The HMBC spectrum of **3**.

**Supplementary Figure S19.** The NOESY spectrum of **3**.

**Supplementary Table S6.** HMBC data of **3**.

| Position | HMBC | Position | HMBC |
| --- | --- | --- | --- |
| 2 | 1, 3, 4, 6,7 | 2' | 1', 3', 4', 6', 7' |
| 5 | 1, 3, 4, 6 | 5' | 1', 3', 4' |
| 6 | 1, 2, 4, 7 | 6' | 1', 4', 7' |
| 7 | 1, 2, 6, 8, 9, 8', 9' | 7' | 8, 9, 1', 2', 6', 8', 9' |
| 8 | 1, 7, 7', 8' | 8' | 7, 8, 1', 7' |
| 9 | 7, 8, 7', 8' | 9' | 7, 8, 7', 8' |
| OCH_3_ | 3 | OCH_2_O | 3', 4' |
| OCH_2_ | 4, CO |  |  |
| COCH_3_ | CO |  |  |


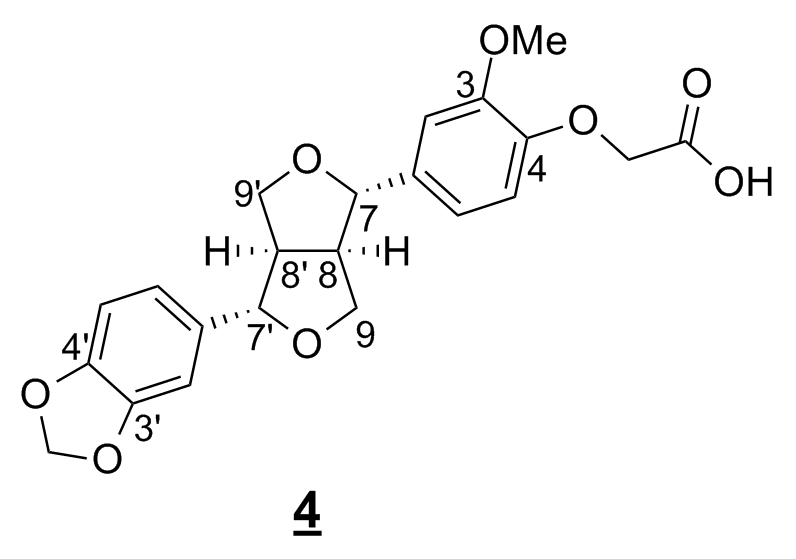

**Supplementary Figure S20.** The ^1^H NMR (acetone-*d*6) spectrum of **4**.

**Supplementary Figure S21.** The ^13^C NMR (acetone-*d*_6_) spectrum of **4**.

**Supplementary Figure S22.** The HSQC spectrum of **4**.

**Supplementary Figure S23.** The HMBC spectrum of **4**.

**Supplementary Figure S24.** The NOESY spectrum of **4**.

**Supplementary Table S7.** HMBC data of **4**.

| Position | HMBC | Position | HMBC |
| --- | --- | --- | --- |
| 2 | 1, 3, 4, 6,7 | 2' | 1', 3', 4', 6', 7' |
| 5 | 1, 3, 4 | 5' | 1', 3', 4' |
| 6 | 1, 4, 7 | 6' | 2', 4', 7' |
| 7 | 1, 2, 6, 8, 9, 8', 9' | 7' | 8, 9, 1', 2', 6', 8', 9' |
| 8 | 1, 7, 7', 8' | 8' | 7, 8, 1', 7' |
| 9 | 7, 8, 7', 8' | 9' | 7, 8, 7', 8' |
| OCH_3_ | 3 | OCH_2_O | 3', 4' |
| OCH_2_ | 4, CO |  |  |
